# An Experimental Study on the Thermal Performance of a Heat Sink Filled with Porous Aluminum Skeleton/Paraffin Composite Phase Change Material

**DOI:** 10.3390/ma17174332

**Published:** 2024-08-31

**Authors:** Shufeng Huang, Zhihan Hu, Zhixin Chen, Dayong Yang, Weili Huang, Bin Zhang

**Affiliations:** 1 School of Mechanical and Electronic Engineering, East China University of Technology, Nanchang 330013, China; 2 School of Mechanical and Automotive Engineering, Guangxi University of Science and Technology, Liuzhou 545006, China

**Keywords:** porous aluminum skeleton, selective laser melting, composite phase change materials, temperature control

## Abstract

Organic phase change material is an ideal solution to solve the heat dissipation problem of electronic devices. However, its low thermal conductivity limits its application. To solve this problem, a new porous aluminum skeleton/paraffin composite phase change material (AS-PCM) was prepared. The effects of porosity and porous aluminum skeletons on temperature control performance were explored. The experimental results show that the addition of AS significantly improves the thermal conductivity of organic PCM, and the thermal conductivity of AS-PCM is 32.3–59.6 times higher than that of pure paraffin. In addition, the temperature difference in AS-PCM with 75% porosity is 1–2 °C lower than that of AS-PCM with 85%, and 5–8 °C lower than that of AS-PCM with 95% porosity. The skeleton structure has an impact on the temperature control performance. The Mcc porous aluminum skeleton/paraffin composite phase change material (MAS-PCM) yields the best thermal performance compared with the Fcc porous aluminum skeleton/paraffin composite phase change material (FAS-PCM). The temperature control time of the MAS-PCM heat sink is increased by 5.3–50.8% relative to the FAS-PCM heat sink. The research results provide a novel approach for improving the thermal conductivity of PCMs.

## 1. Introduction

With the rapid development of the microelectronic technology industry, various electronic components such as batteries, light-emitting diodes (LEDs), and photovoltaic modules are becoming highly efficient, lightweight, and miniaturized. However, this leads to heat dissipation problems [1,2,3]. Therefore, to keep the temperature of electrical components below the critical limit, effective cooling technology is required. The conventional water cooling method is very effective for thermal management, but this approach does not seem to be a viable option for electronic components because this may require additional power consumption and extra space due to the large size [4].

PCMs exhibit good chemical stability, large latent heats, and non-toxicity; as a result, PCM is used in a variety of industrial and engineering applications [2] such as photovoltaic modules, battery thermal management, solar air heaters, and thermal comfort in buildings and food packaging [5]. PCM may be suitable for the thermal management of interstitial work electronics. However, the poor thermal conductivity of organic PCMs limits their applications [6,7]. To deal with the issue, various techniques have been proposed to improve thermal conductivity. Among these methods, embedding fins, using high-conductivity composite PCMs, and introducing porous metallic skeletons have been widely used [8]. Embedding fins can improve the efficiency of heat exchange from the heat source to the PCM while increasing the response time of PCMs [9,10,11]. However, embedding fins have many disadvantages, such as increased system mass, volume, and material consumption [12]. Dispersing high-conductivity nanomaterials is an effective method to improve thermal conductivity of PCMs. Many researchers have dispersed high-conductivity nanofillers into PCMs to prepare composite PCMs with high thermal conductivity [7,13,14]. However, there ae two drawbacks [12]: the first is that nanofillers reduce the viscosity of liquid PCMs and weaken the natural convection [15], and the second is that the long-term use of nanofillers may cause sedimentation and deposition [16].

Embedding PCMs into porous materials has attracted widespread attention as a method of improving their thermal conductivity [17,18]. Expanded metal mesh, graphite matrix, powder porous structure, and metal foam are commonly used materials. Mustaffar et al. [19] prepared an expanded metal mesh/PCM and showed that it resulted in an 81% reduction in melting time, outperforming metal foam/PCM. However, it is necessary to solve the problem of large contact thermal resistance between metal mesh layers. Graphite matrices/PCM was prepared by Mills et al. [20]. The results showed that the addition of porous graphite achieved an increase of two orders of magnitude in the thermal conductivity of PCM. However, the thermal conductivity of their PCM is anisotropic. Li et al. [6] studied the thermal behavior of copper-powder-sintered frame/paraffin PCMs. The fluidity of liquid PCMs in powder form is poor. Many scholars have studied another type of foam porous composite energy storage material, demonstrating that it can improve thermal conductivity, homogenize the internal temperature distribution, and shorten the melting time of PCMs [21,22]. However, foamed metal materials are expensive to manufacture and have closed pores [23], limiting their large-scale application. Porous stainless steel fibers [24] have been used to improve the thermal conductivity of PCM; however, their smooth surface prevents them from fully improving heat exchange between PCMs and porous fibers. Porous copper fiber/PCM [23] was developed because of its main advantages of a three-dimensional (3D) mesh and good connectivity structure. However, this type of porous copper fiber with high porosity is difficult to manufacture.

According to the above analysis, the internal pores of the above-mentioned porous structure have random, uncontrollable, closed and inconsistent pore sizes, which are fatal defects for applications. Therefore, designing and manufacturing suitable porous skeleton applications for PCM with the high thermal conductivity remains a challenge. In addition, there is little research on the influence of porosity and the skeleton structure of porous skeleton composite phase change material on electronic heat dissipation. To fill this research gap, a novel porous aluminum skeleton (AS) was designed and manufactured using a selective laser melting method. And porous aluminum skeleton/paraffin composite phase change material (AS-PCM) was prepared. The influence of porosity and skeleton structure of AS-PCM on the thermal control of electronic devices was experimentally investigated.

## 2. Preparation and Experimental Setup

### 2.1. AS-PCM Heat Sinks with a LED Light Source

**Figure 1:**
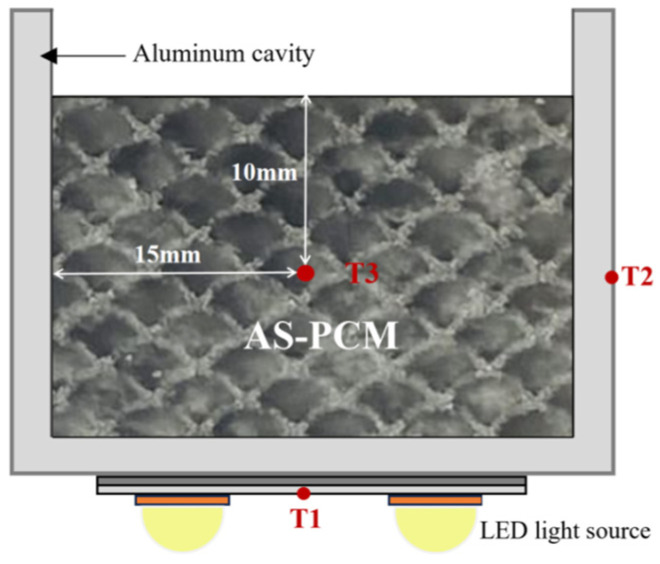
AS-PCM heat sink.

As shown in Figure 1, the diagram of the AS-PCM heat sink includes an aluminum cavity of cuboid structure, AS-PCM, and a light-emitting diode (LED) light (Cree lnc., Durham, NC, USA). The heat storage function of the heat sink is mainly realized by the internal AS-PCMk. The aluminum cavity size is as follows: a wall thickness of 2 mm and inner cavity size of 30 mm × 30 mm × 25 mm. The LED light was fixed to center of the bottom surface of the cavity with a heat-conducting double-sided tape, and the power range of the LED light was 6–30 W. As shown in Figure 1, the AS-PCM heat sink has three temperature measuring points at the bottom (T1), side (T2), and the center of the AS-PCM (T3). 

### 2.2. Preparation of AS-PCM

**Figure 2:**
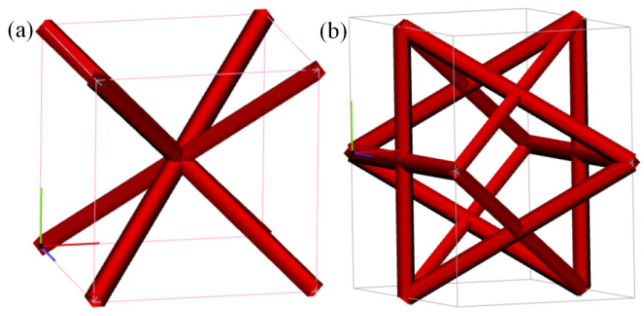
Architecture single cell: (**a**) Mizi center cubic, (**b**) Face center cubic.

**Figure 3:**
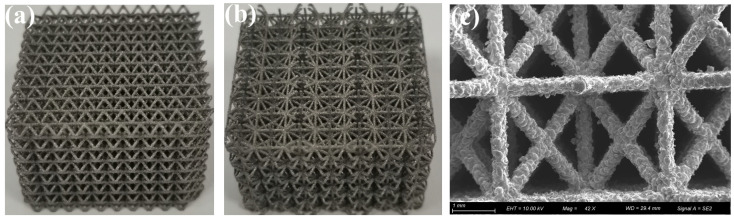
Porous aluminum skeleton: (**a**) M85AS, (**b**) F85AS, (**c**) the SEM image of M85AS.

The AS-PCM was prepared in two steps: the design and fabrication of AS using the selective laser melting method and the preparation of AS-PCM based on the water bath method. As shown in Figure 2, the architecture single cells for the Mizi center cubic (Mcc) and Face center cubic (Fcc) are designed, and then the Mcc porous aluminum skeleton (MAS) and the Fcc porous aluminum skeleton (FAS) are designed by arraying and filling method. AlSi10Mg aluminum alloy material was selected, and MAS and FAS were fabricated based on the selective laser melting (SLM) method. Figure 3a,b shows the MAS with 85% porosity (M85AS) and the FAS with 85% porosity (F85AS). Figure 3 illustrates that FAS and MAS have a three-dimensional regular arrangement of holes, excellent connectivity, and a rough surface, promoting the natural flow of liquid paraffin between porous AS. For each porosity of the AS samples, the mass volume method is used to determine the following:

(1) $$\varepsilon =\left(1-\frac{m_{Al}}{\rho_{Al}V}\right)$$

where *V* is the volume of the AS (m^3^), $m_{Al}$ is the mass of AS (kg), and $\rho_{Al}$ is the density of Al (kg/m^3^).

The PCM material for this experiment is paraffin wax produced by RUBITHERM, Berlin, German. Normal paraffin of type C_n_H_2n+2_ is recommended. The thermal properties of the PCM are presented in Table 1.

**Figure 4:**
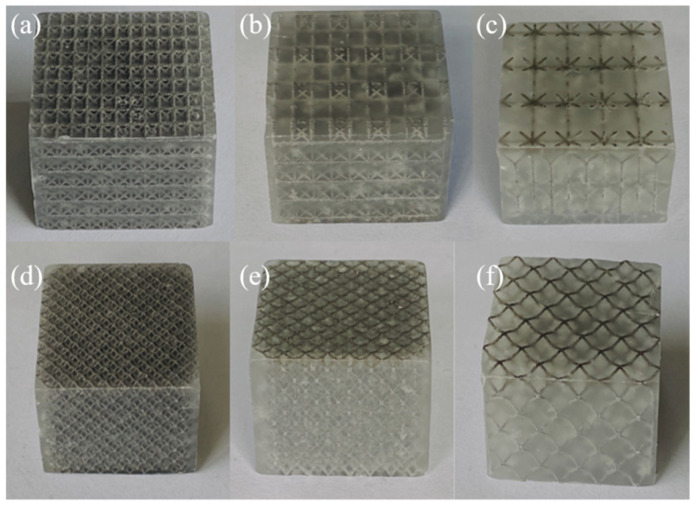
AS-PCM samples: (**a**) F75AS-PCM, (**b**) F85AS-PCM, (**c**) F95AS-PCM, (**d**) M75AS-PCM, (**e**) M85AS-PCM, (**f**) M95AS-PCM.

AS-PCMs were prepared based on the water bath method. The steps were as follows: (1) Place a heat-resistant soft silicone mold into a beaker, weigh an appropriate amount of paraffin wax into the silicone mold, and place the AS on top of the paraffin wax. (2) Place the beaker in the water bath and allow the paraffin to liquefy and the AS intrude into the paraffinic liquid. (3) Keep the water bath at 80 °C for some time, turn off the water bath and allow the sample to cool naturally until the paraffin in the beaker solidifies. The residual paraffin on the surface of the sample was removed, and the final size was adjusted to 30 mm × 30 mm × 20 mm, as shown in Figure 4.

Based on the transient planar heat source method, the thermal conductivity of the samples was tested using the DRE-III thermal conductivity testing instrument, as seen in Table 2. The accuracy of the thermal conductivity is ±0.03 W/m K. For each sample, 6 different points were tested for the thermal conductivity and the average value was taken. The results show that the thermal conductivity of the M95AS-PCM is higher than that of the F95AS-PCM at the same 95% porosity. Meanwhile, the thermal conductivity of MAS-PCM increases with decreasing porosity.

### 2.3. Experimental Setup

**Figure 5:**
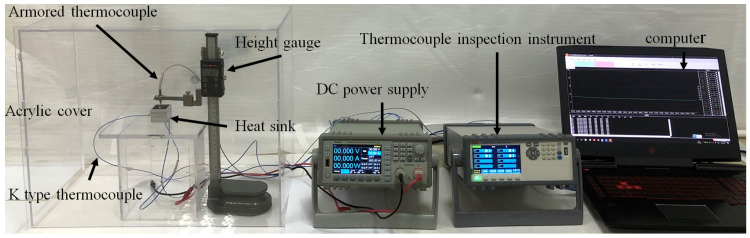
Physical diagram of the experimental setup.

Figure 5 describes an image of the experimental setup, which contains a heat sink with an LED, a DC power supply (HSP-4520, Shenzhen Henghuiyuan Electronics Co., Ltd. Shenzhen, China), a temperature inspection instrument, a height gauge, a laptop computer, and an acrylic cover. Heat sinks were placed in acrylic boxes to prevent heat exchange with the surrounding environment. The two K-type thermocouples were glued to the center of the LED baseplate (T1) and the side center of the cavity (T2) using a thermocouple test adhesive to collect their instantaneous temperatures. Another thermocouple that measured the center temperature (T3) was fixed and precisely positioned using the height gauge. The LEDs were powered via a DC power supply, and four power were used for each heat sink, which were 11.7 W, 14.7 W, 17.7 W, and 21.3 W. The experimental process is as follows: the input power is pre-adjusted, the power supply is pushed on, and the temperature acquisition software (JK5080_V1.2.) is started. When T1 reaches 90 °C or 100 °C, the DC power supply is cut off to stop the heating process. After the heat sink cools naturally to room temperature, data collection is stopped. The K-type thermocouples is made by ourselves and calibrated by the standard platinum resistance. The accuracy of K-type thermocouples and the input power is 0.1 °C and ±0.001 W, respectively.

## 3. Results and Discussion

### 3.1. Temperature Control Performance of a Typical AS-PCM Heat Sink

**Figure 6:**
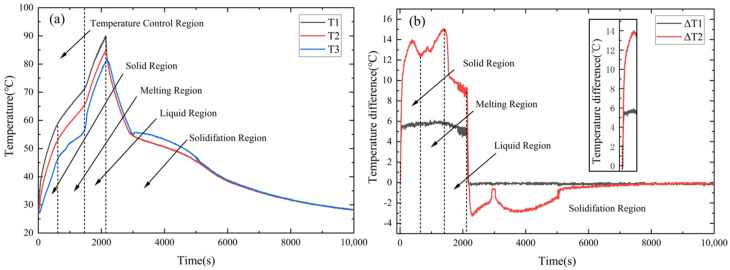
(**a**) Typical transient temperature and (**b**) temperature difference curve of the M95AS-PCM heat sink.

Figure 6 shows the temperature control performance of a typical M95AS-PCM heat sink at 11.7 W. According to Figure 6a, the temperature rise rate curve of the M95AS-PCM can be separated into four phases: solid, melting, liquid, and solidification regions. The average temperature rise rate *V* of T1 is calculated by the following equation:

(2) $$V=\frac{\Delta T}{\Delta \theta }$$

where Δ*θ* is the duration of the corresponding interval and Δ*T* is the temperature rise in T1 during period Δ*θ*.

Figure 6b depicts the temperature difference curve of the M95AS-PCM heat sink. ΔT1 and ΔT2 are defined as ΔT1 = T1 − T2 and ΔT2 = T1 − T3, respectively. The thermal characteristic of the M95AS-PCM heat sink is analyzed as follows:

In the solid-state phase, T1 temperature rises linearly at first, then drops sharply at 565 s. When the LED light source is powered on, ΔT2 increases sharply, reaching a maximum value of 14 °C at 325 s and dropping to 12.7 °C at 720 s. Therefore, the first trough indicates whether it has entered the melting region. In the melting state region, the rate of temperature rise slows considerably, as seen in Figure 6a. ΔT1 has little near-horizontal fluctuation during the melting phase. However, ΔT2 continues to increase, reaching a maximum of 15 °C, because the AS-PCM material acts as a latent heat storage function. In the liquid state region, the temperature rise rate increases again, and the temperature rise slope of T3 is greater than that of T1 and T2. In addition, when ∆T1 enters the liquid state region, it changes from an upward trend to a downward trend. When T1 reached 90 °C, the DC power supply was turned off, and the AS-PCM entered the solidification state region. T2 and T1 immediately decreased at the same rate, while T3 increased from 80.8 °C to 82 °C in the first 40 s and then rapidly decreased, as shown in Figure 6a. Afterward, the cooling rates of the three temperature measurement points simultaneously turned and slowed down as the AS-PCM began to solidify. The AS-PCM material was completely solidified at 5505 s, and the temperature dropped to 27 °C.

The studies mentioned above demonstrate that the AS-PCM loses its phase transition temperature control capability once it reaches the liquid region. Considering this, only the solid-state and melting-state stages are used for the temperature control of electronic devices. Therefore, the working time of the electronic device is less than the temperature control times of the AS-PCM, and the non-working time is greater than the solidification region time of the AS-PCM in engineering applications.

### 3.2. Influence of Different Heat Sink Types during the Heating Stage

**Figure 7:**
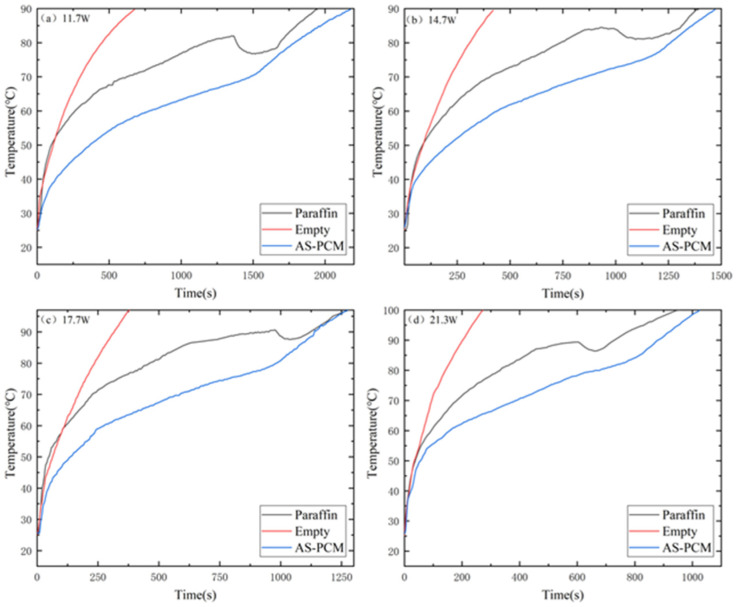
Comparison of transient temperature control performance of the three types: (**a**) 11.7 W, (**b**) 14.7 W, (**c**) 17.7 W and (**d**) 21.3 W.

Figure 7 compares T1 temperature control performance under the three heat sink types (empty heat sink, paraffin heat sink, and AS-PCM heat sinks). For an empty heat sink, the heat is mainly transmitted to the cavity surface through heat conduction and weakly natural convection into the environment. Consequently, T1_Empty_ rises rapidly, reaching a critical temperature of 90 °C at 11.7 W in 685 s, far exceeding that of paraffin and AS-PAM. This proves that the lack of PCMs can lead to thermal control problems in electronic devices. In addition, a localized once-drop appears in the temperature curve due to the presence of a small suspended solid paraffin block. The solid paraffin block rapidly absorbs heat and melts, which is also observed at other input powers.

For the AS-PCM heat sink, the temperature rise slope of T1_AS-PCM_ is much slower than that of T1_Paraffin_, and the duration of the solid phase is extended by 700 s before the melting stage begins. At this moment, T1_AS-PCM_ is 12 °C lower than T1_Paraffin_. This is because the thermal conductivity of AS-PCM is 32.2~59.6 times higher than that of pure paraffin. During the melting region, T1_AS-PCM_ also rises at a slightly lower speed than T1_paraffin_. This is attributed to the reason that the effect of increased thermal conductivity is greater than the effect of inhibiting natural convection. Therefore, the T1_AS-PCM_ of AS-PCM is lower.

**Figure 8:**
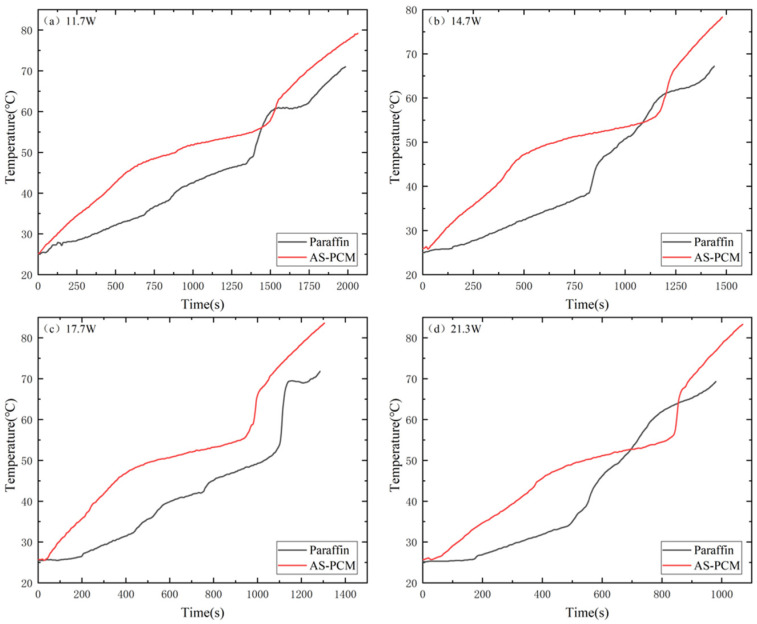
Change in central temperature (T3) of phase change material at heating stage under different input power: (**a**) 11.7 W, (**b**) 14.7 W, (**c**) 17.7 W and (**d**) 21.3 W.

To further understand the melting process of AS-PCM and paraffin types, temperature variations (T3 and ΔT2) may be compared. As shown in Figure 8, T3_paraffin_ remained below T3_AS-PCM_ under the four input powers before entering the melting zone, and the rising slope of T3_AS-PCM_ was larger than that of T3_paraffin_ in the solid phase region, indicating that the heat could be transferred to AS-PCM faster due to the high thermal conductivity of AS. In addition, it can be seen from Figure 8 that T3_paraffin_ appears as an inflection point and the temperature rises rapidly, which is caused by the rapid melting and absorption of heat by the small solid paraffin float in the middle. This phenomenon corresponds to the concave of T1_paraffin_ in Figure 7.

**Figure 9:**
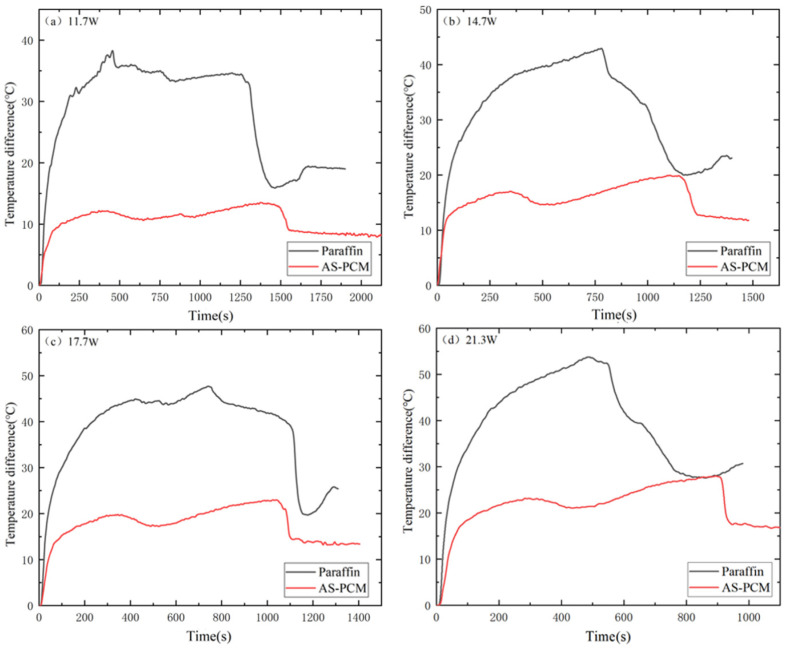
Temperature difference change in phase change materials at the heating stage under different input power (ΔT2) changes: (**a**) 11.7 W, (**b**) 14.7 W, (**c**) 17.7 W and (**d**) 21.3 W.

As shown in Figure 9, ΔT2_AS-PCM_ is much smaller than ΔT2_paraffin_ at any moment of all four input powers. When entering the liquid phase, the temperature difference between the two types is approximately 25–30 °C, which is approximately 2.5 times that of ΔT2_AS-PCM_. This shows that the existence of AS greatly improves the heat exchange efficiency of paraffin, making the temperature change uniform. Therefore, it is better to consider the AS-PCM heat sink for the thermal control of electronic devices in engineering applications.

### 3.3. Influence of Porosity of MAS-PCM during the Heating Stage

**Figure 10:**
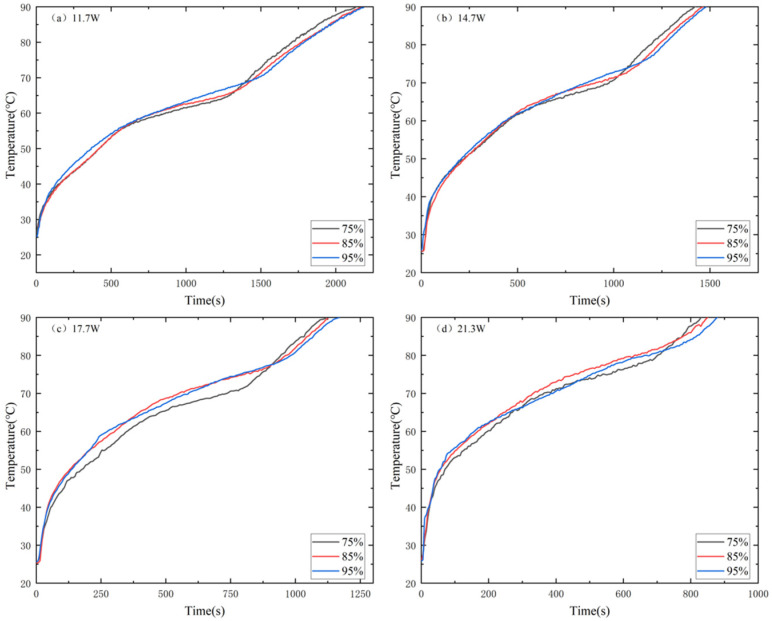
Comparison of different power T1 at the heating stage of MAS-PCM with different porosities: (**a**) 11.7 W, (**b**) 14.7 W, (**c**) 17.7 W and (**d**) 21.3 W.

Figure 10 depicts the effect of porosity on T1 of the MAS-PCM heat sink. In the solid state, T1_75%_ < T1_85%_ < T1_95%_. In the melting zone, the T1 of the MAS-PCM with 75% porosity was smaller than that of the MAS-PCM with 85% and 95% porosity, and the temperature difference between the three porosity MAS-PCMs gradually increased. This is because the natural convection is more inhibited in the MAS-PCM with low porosity, owing to low permeability. And the heat conduction enhancement by the MAS-PCM with low porosity exceeded the inhibition of paraffin natural convection.

From another point of view, in the case of a fixed diameter, the aperture and interface area of the porous medium can be defined as follows [26]:

(3) $$\phi_{sf}=\frac{2\sqrt{3\pi (1-\varepsilon )}}{d_{ep}}=\frac{3\pi d_{f}}{{d_{ep}}^{2}}$$

Therefore, the smaller the porosity of MAS, the larger its specific surface area. Thus, the thermal connectivity between the paraffin and MAS is stronger at a smaller porosity, resulting in a smaller T1 at lower porosity.

The higher the porosity, the longer the duration of the temperature control zone. This is because the MAS-PCM with higher porosity has more paraffin. Due to the sharp increase in T1 after complete melting, T1_75%_ ultimately reaches its peak in the liquid stage. Before the complete melting of the MAS-PCM, the difference between the three porosities increases with the increase in power, indicating that materials with low porosity perform better in reducing the higher power of T1 before complete melting.

**Figure 11:**
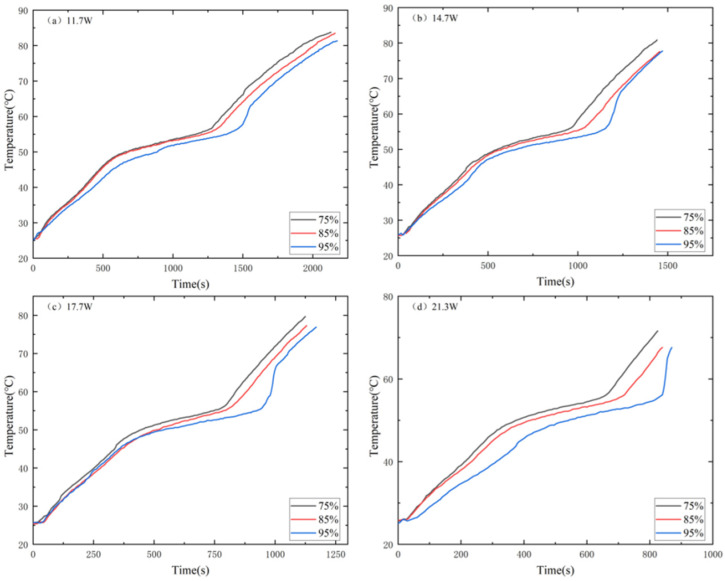
T3 comparison of MAS-PCM with different porosity: (**a**) 11.7 W, (**b**) 14.7 W, (**c**) 17.7 W and (**d**) 21.3 W.

**Figure 12:**
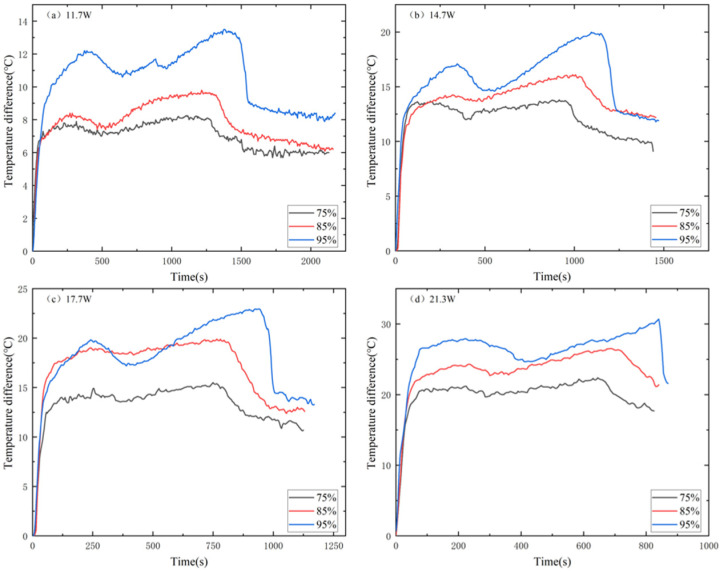
Temperature difference comparison of MAS-PCM with different porosities: (**a**) 11.7 W, (**b**) 14.7 W, (**c**) 17.7 W and (**d**) 21.3 W.

Figure 11 shows the effect of MAS porosity on T3. The T3 of low-porosity MAS-PCM is higher than that of large-porosity MAS-PCM, and the T3 difference between the three types of porosity becomes more evident as the power increases before entering the liquid phase. At the same power, the MAS-PCM with low porosity has a higher T3 and more heat is transferred to the MAS-PCM with low porosity. In addition, the melting duration of the MAS-PCM with low porosity is shorter than that with high porosity, indicating that the larger the porosity, the larger the temperature control interval. Figure 12 shows the effect of porosity on the temperature difference ΔT2. At any input power, ΔT2 with low porosity is smaller than ΔT2 with high porosity. Before complete melting, using 11.7 W as an example, ΔT2_75%_ is 1–2 °C lower than ΔT2_85%_ and 5–8 °C lower than ΔT2_95%_. This indicates that the temperature gradient of low-porosity MAS-PCM is smaller in the temperature control zone, which is beneficial for the low-temperature operation of electronic devices.

### 3.4. Influence of the Skeleton Structure of AS-PCM during the Heating Stage

**Figure 13:**
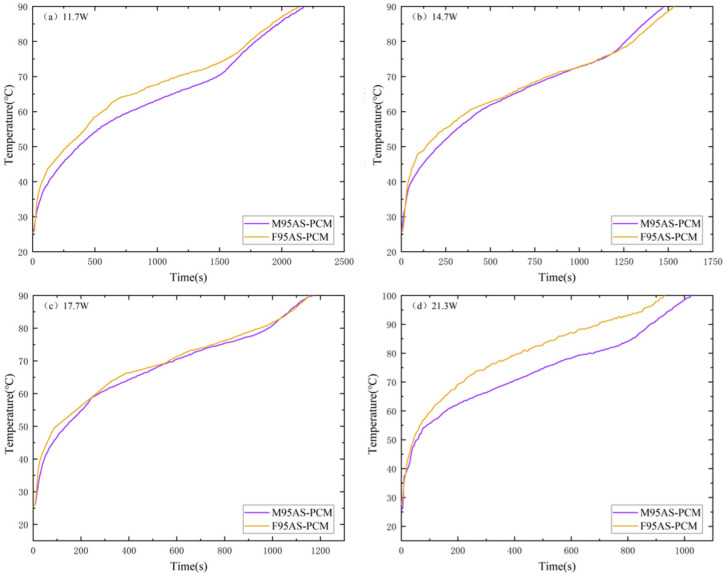
Heating stage T1 of AS-PCM with two kinds of aluminum skeleton structures: (**a**) 11.7 W, (**b**) 14.7 W, (**c**) 17.7 W and (**d**) 21.3 W.

Figure 13 depicts the influence of the aluminum skeleton structure (MAS and FAS) on T1. AS-PCM with 95% porosity was used for comparative analysis. Before the first three powers, the heating rate of F95AS-PCM was higher than that of M95AS-PCM in the solid phase. In the melting phase, the heating rate of the two structures was almost the same, whereas in the liquid phase, the heating rate of M95AS-PCM was slightly higher than that of F95AS-PCM. At a maximum of 21.3 W, the internal paraffin can be completely liquefied before 90 °C, whereas FAS-PCM maintains the heating rate of the melting stage until 90 °C, at which point the internal paraffin is not completely liquefied, affecting the temperature control effect, and T1_FAS_ > T1_MAS_. This can be attributed to the following reasons: the thermal conductivity of M95AS-PCM is 4.3% higher than that of F95AS-PCM. Therefore, the T1 of M95AS-PCM was lower than that of F95AS-PCM in the melting region.

**Figure 14:**
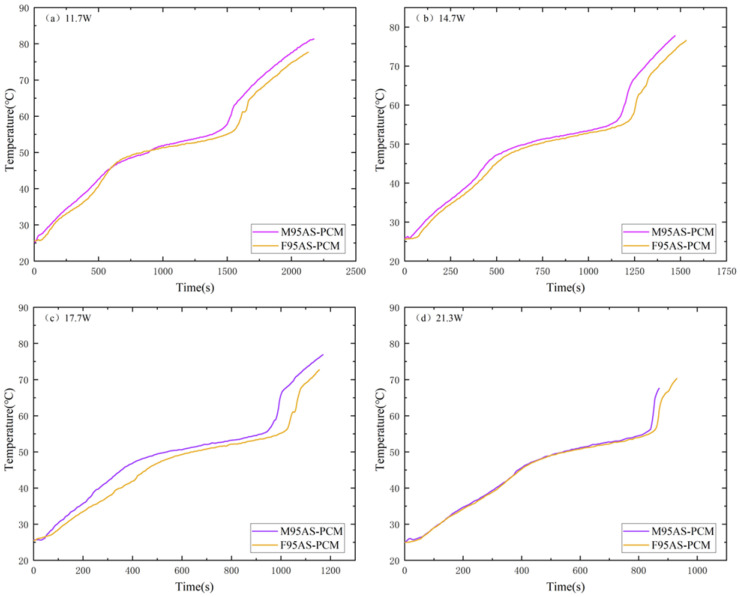
Heating stage T3 of AS-PCM with two types of aluminum skeleton structures: (**a**) 11.7 W, (**b**) 14.7 W, (**c**) 17.7 W and (**d**) 21.3 W.

**Figure 15:**
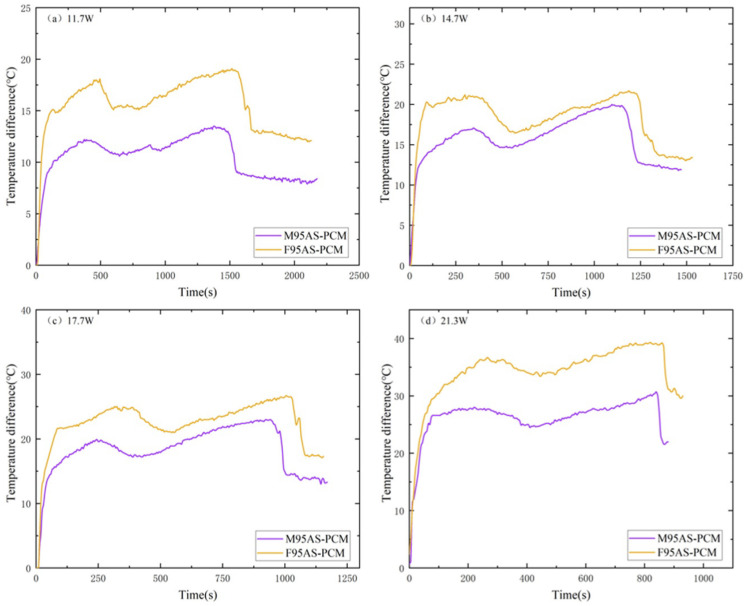
Heating stage of AS-PCM with two aluminum skeleton structures, ΔT2: (**a**) 11.7 W, (**b**) 14.7 W, (**c**) 17.7 W and (**d**) 21.3 W.

Figure 14 shows the T3 comparison of AS-PCM heat sinks with two structures in the heating stage. The temperature curve of AS-PCM is T3_FAS_ > T3_MAS_. The high heat flow at 21.3 W makes the F95AS-PCM unable to effectively transmit the bottom temperature to the center; therefore, its final center temperature is far lower than the center temperature of M95AS-PCM. Figure 15 shows the influence of two skeleton structures under the influence of ΔT2. The AS-PCM of the two aluminum skeleton structures under the four powers are ΔT2_FAS_ > ΔT2_MAS_. Using 11.7 W as an example, ΔT2 is lower than that of the FAS structure, by about 4–6 °C. Thus, it is shown that the structure of MAS-PCM aids the device in maintaining a low operating temperature.

### 3.5. Heat Sink Performance during the Cooling Stage

**Figure 16:**
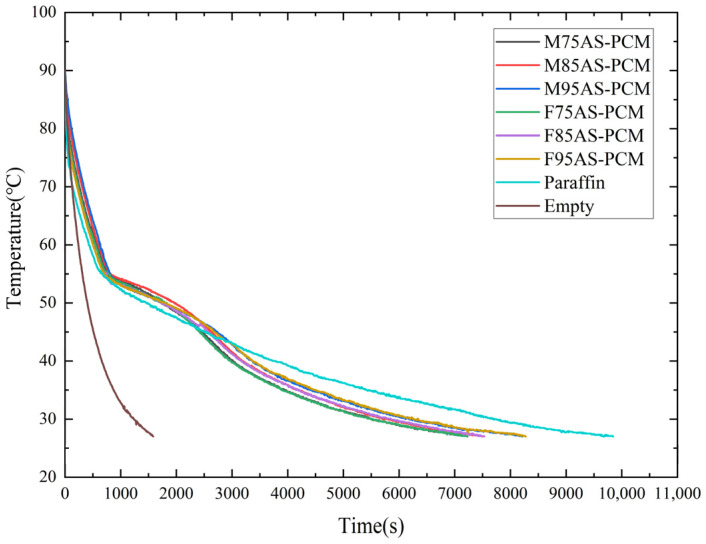
Cooling curves of AS-PCM with three different types and porosities.

Figure 16 shows the cooling curve of all heat sink T1 points from 90 °C to 27 °C. The results show that the cooling rate of both structures is the fastest at low porosity. Under the same porosity, the cooling time of MAS-PCM is shorter, and the advantage is more obvious in the case of high porosity. The time from the beginning of cooling to the complete solidification of paraffin in both structures is T1_95%_ < T1_85%_ < T1_75%_ because the path of heat transfer in the cooling process is from the internal PCM to the heat sink shell, and the paraffin stores more heat. The higher the paraffin content, the longer the solidification. As for the effect of the structure on the cooling rate, since the specific surface area of MAS is larger than that of FAS, the heat is exported faster, resulting in a shorter cooling time for MAS-PCM than for FAS-PCM.

### 3.6. Influence of Different AS-PCM on Temperature Control Time

**Figure 17:**
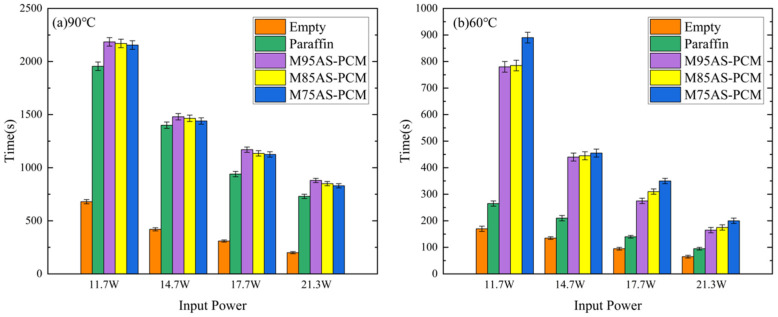
Temperature control time diagram of different types and porosities: (**a**) 90 °C, (**b**) 60 °C.

Figure 17 shows that the PCM-based heat sinks have significantly longer temperature control times than empty heat sinks, which indicate that the introduction of PCM makes the electronic device operate longer at low temperatures. In addition, whether the critical temperature is 60 °C or 90 °C, the temperature control time of the paraffin heat sink is much shorter compared with the AS-PCM heat sink. Taking M95AS-PCM as an example, when the critical temperature is 90 °C, the temperature control time of the M95AS-PCM heat sink is increased by 5.7–20.5% compared with that of pure paraffin heat sink. When the critical temperature is 60°C, the temperature control time of the M95AS-PCM heat sink is 1.73–2.94 times higher than that of the pure paraffin heat sink.

**Figure 18:**
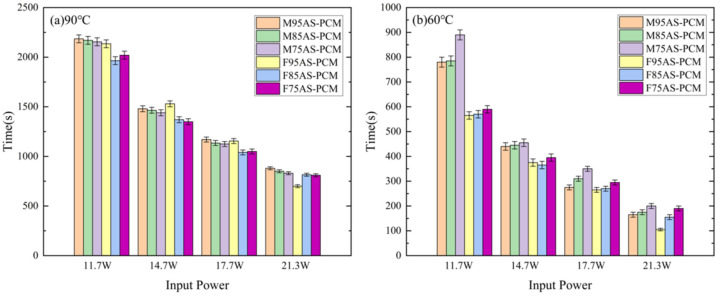
Temperature control time diagram of different AS-PCMs: (**a**) 90 °C, (**b**) 60 °C.

From Figure 18a, it can be seen that as the porosity increases, the temperature control time of the two skeleton structures increases under the critical temperature of 90 °C. Meanwhile, the MAS-PCM has the best temperature control performance at a porosity of 95%. Figure 18b shows that regardless of the power, the temperature control time of the MAS-PCM is longer than that of the FAS-PCM under the same porosity. However, as the power increases, the difference in temperature control time between the two structures gradually decreases. Taking M85AS-PCM and F85AS-PCM as examples, when the critical temperature is 60 °C, the temperature control time of M85AS-PCM is increased by 12.9–37.7% compared with that of F85AS-PCM. Therefore, in practical applications, the MAS-PCM with low porosity should be selected based on the power requirement of the LED light and the critical temperature.

### 3.7. Comparison with Other Reported PCMs

In order to better evaluate the thermal conductivity of the new AS-PCM, a comparison was made between the new AS-PCM and other PCMs reported in the literature, as shown in Table 3. The maximum thermal conductivity of AS-PCM is 4.2, 1.59, 11.92 and 1.19 times higher than that of graphite based nanocomposites–PCM [27], aluminum foam–PCM [28], graphite matrix–PCM [29] and copper foam–PCM [30], respectively. Therefore, the new AS-PCM yields the best thermal conductivity.

## 4. Conclusions

(1) A new porous aluminum skeleton was proposed to solve the low thermal conductivity problem of organic PCM. Compared with organic PCM, the thermal conductivity of the new AS-PCM is 32.2~59.6 times higher than that of pure paraffin.
(2) The lower the porosity of the AS, the faster the cooling speed, and the MAS-PCM heat sink enables the LED device to operate at a lower temperature.
(3) At a critical temperature of 90 °C, the temperature control time of M95AS-PCM is longer than that of others. The temperature control time of the M95AS-PCM heat sink is increased by 5.7–20.5% compared with that of the pure paraffin heat sink. At the critical temperature of 60 °C, the temperature control time of the M75AS-PCM heat sink is increased by 5.3–50.8% compared with that of F75AS-PCM. The new AS-PCM is a promising and reliable material for the thermal control of electronic devices.

## Figures and Tables

**Table 1:** Thermal properties of paraffin wax [25].

*T_m_* (°C)	*K* (W/m·K)	*ρ* (kg/m^3^)	*C_p_* (kJ/kg)	Heat Storage Capacity (kJ/kg)
56–58	0.2	880/790	173.6	2.8

**Table 2:** Thermal conductivity of AS-PCMs.

AS-PCM	M75AS-PCM	M85AS-PCM	M95AS-PCM	F75AS-PCM	F85AS-PCM	F95AS-PCM
*K* (W/m·K)	11.92	7.54	6.73	11.45	7.29	6.45

**Table 3:** Comparison between the present study and other reported PCMs.

Authors	Configuration	kpcm (W/m·K)	PCM
Present study	AS-PCM	6.45–11.92	Paraffin
Farid et al. [27]	Graphite-based nanocomposites–PCM	2.8	Paraffin
Lafdi K. et al. [28]	A luminum foam–PCM	7.5	Paraffin
Salma Gharbi et al. [29]	Graphite matrix–PCM	1	Paraffin
Kothari R. et al. [30]	Copper foam–PCM	10	Paraffin

## Data Availability

All data to support the results of this study are included in the article.

## Nomenclature

Nomenclature			
*C_p_*	heat capacity (kJ/kg)	*AS*	porous aluminum skeleton
*V*	volume (m^3^)	*MAS*	Mcc porous aluminum skeleton
*m*	mass (kg)	*FAS*	Fcc porous aluminum skeleton
*T*	temperature	*AS-PCM*	aluminum skeleton/paraffin composite
Δ*T*	temperature difference	Greek symbols	
*T_m_*	paraffin melting temperature	*ε*	porosity (%)
*K*	thermal conductivity (W/m·K)	*ρ_Al_*	density of aluminum skeleton
*d_ep_*	wire diameter (m)	*ρ*	paraffin wax density (kg/m^3^)
*m_Al_*	weight of aluminum skeleton (kg)	Δ*θ*	time (s)
Abbreviations		*ϕ_sf_*	specific surface area
*PCM*	phase change material		
